# Laparoscopic Collis-Nissen Procedure in a Patient With Type IV Hiatal Hernia: Relevance of a Short Esophagus

**DOI:** 10.7759/cureus.67530

**Published:** 2024-08-22

**Authors:** Carlos F Acuña Cota, Jorge L Bravo Raygoza, Christian Ballardo Medina, Jaime Matus Rojas, Maria V Figueroa Beltran

**Affiliations:** 1 General Surgery, Instituto de Seguridad y Servicio Social de los Trabajadores del Estado, Culiacán, MEX

**Keywords:** nissen fundoplication, collis gastroplasty, short esophagus, fundoplication, hiatal hernia, laparoscopic hernioplasty

## Abstract

A short esophagus is generally diagnosed during antireflux surgery and is defined as a distance of less than 2 cm between the gastroesophageal junction and the apex of the hiatus. We present a female patient with a CT diagnosis of type IV hiatal hernia who was scheduled for antireflux surgery, showed a short esophagus during the procedure, opted to perform Collis gastroplasty, and discharged without complications. A short esophagus remains a controversial topic. Some authors argue that it is more common than suspected and responsible for high recurrences in specific patient groups.

## Introduction

A true short esophagus is diagnosed during hiatal hernia surgery when the distance between the gastroesophageal junction and the apex of the hiatus is less than 2 cm after maximum mobilization of the distal esophagus without tension toward the stomach. It is found in up to 20% of patients receiving routine surgery for gastroesophageal reflux disease (GERD) and in more than 50% of type III-IV hiatal hernias. Currently, Collis gastroplasty (CG) with fundoplication is the standard treatment in patients who, after extensive dissection of the distal esophagus, have a short esophagus [1,2]. In our case, the hernial component is comprehensively addressed with abundant paraesophageal dissection as well as gastroplasty according to the length of the intra-abdominal esophagus, obtaining a favorable evolution in the short and medium term without symptoms of reflux at the moment.

## Case presentation

**Figure 1:**
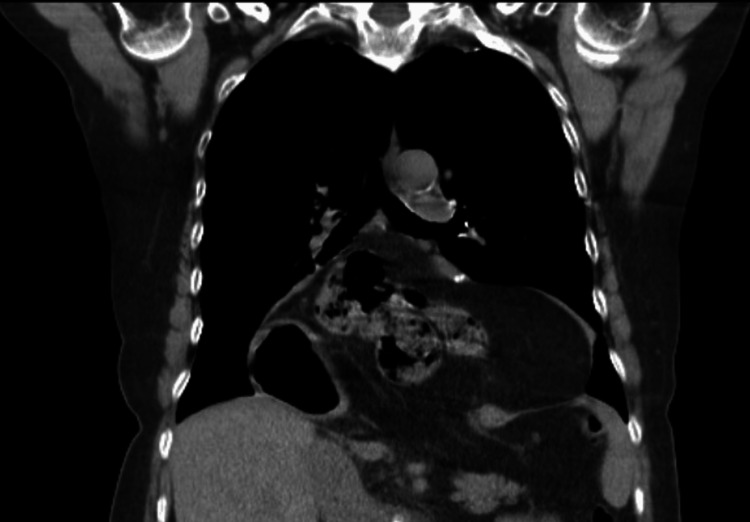
Type IV hiatal hernia demonstrated by CT CT: computed tomography

A 63-year-old female, diabetic and hypertensive, presented with a current condition that begins with heartburn and has been going on for years. For five months, her heartburn has worsened and does not respond to the intake of omeprazole and dietary hygiene actions, accompanied by respiratory difficulty. A CT scan of the chest was performed, which showed the passage of abdominal contents into the thoracic cavity (Figure 1).

**Figure 2:**
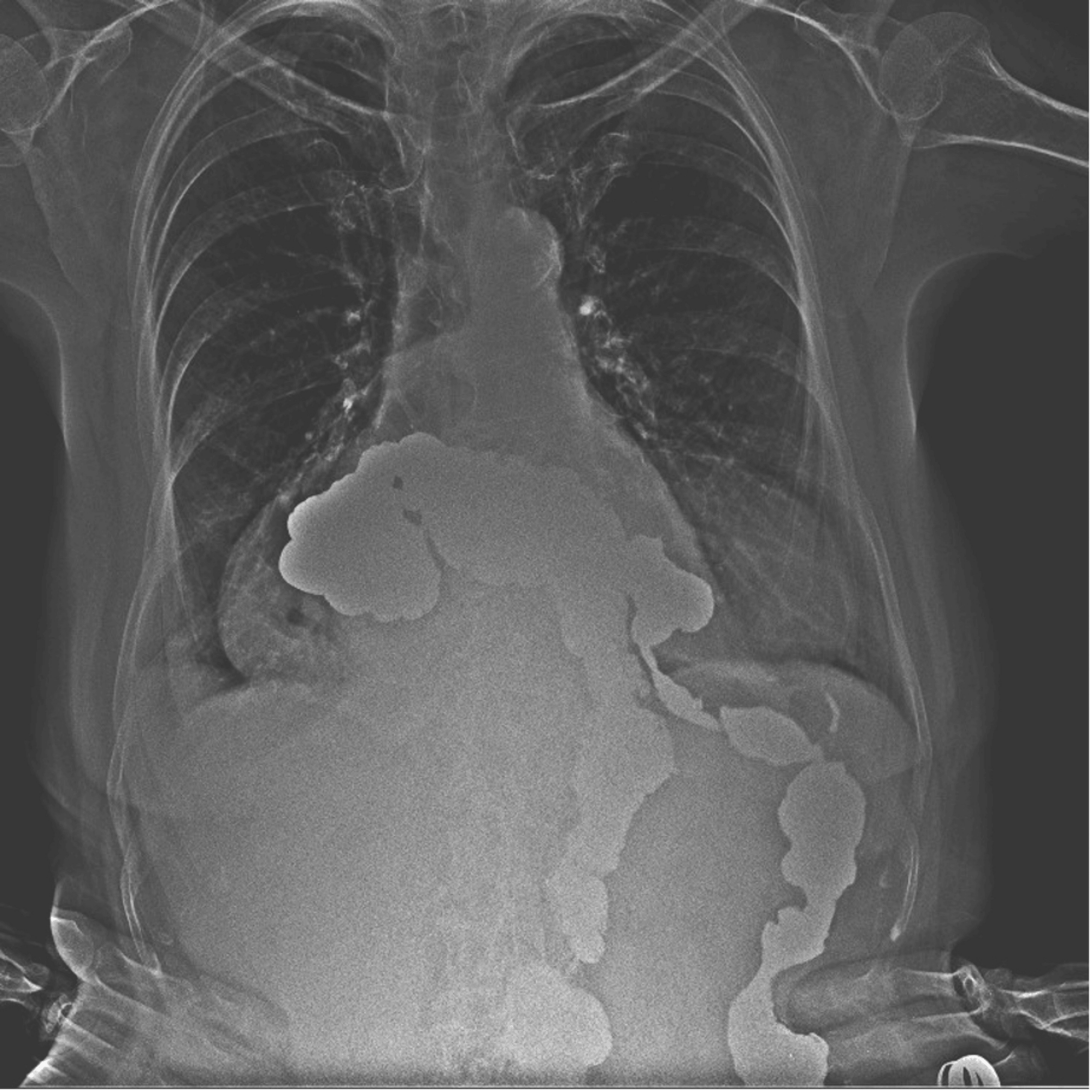
Type IV hiatal hernia demonstrated by esophagogram Note the distortion of the stomach shape due to its intrathoracic situation

She was referred to general surgery, and a study protocol was initiated, including an esophagogram (Figure 2) and an endoscopy. The endoscopy reported inflammatory peptic-like stenosis that prevents the passage of the endoscope at 37 cm from the dental arch. It was dilated with a balloon for one minute without reaching the distal esophagus. A chest CT showed a hernia with intestinal loops including the entire stomach; the hernia sac measured 112.6 x 174.2 mm.

**Figure 3:**
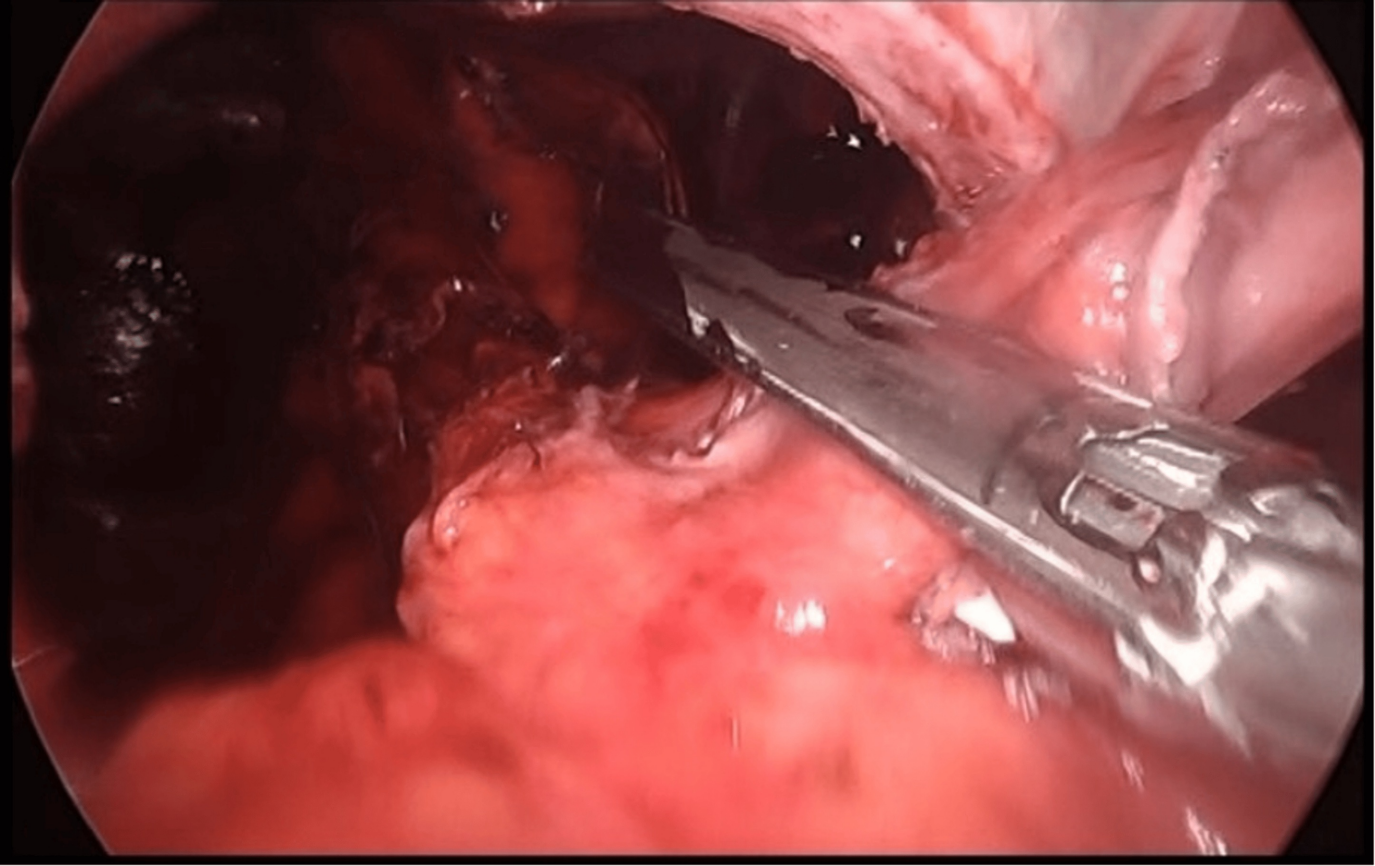
Fundectomy during CG CG: Collis gastroplasty

It was decided to schedule a laparoscopic paraesophageal hernia repair (Figure 3). The procedure involved locating the thoracic esophagus, gastric, and intestinal components. The hernia sac was dissected and exposed, and the complete component was released into its thoracic cavity. Short vessels and the phrenoesophageal membrane were dissected until the pillars of the hiatus were identified. Perihiatal ligament dissection was performed using LigaSure (Medtronic, Dublin, Ireland) exposing the two pillars. The retroesophageal space was created, and a wedge fundoplication was performed using 60 mm staples until the esophagus was elongated by 2 cm posterior to the pillars, creating an esophagogastric neounion. The gastric fundus was rotated 360° and fixed to the right pillar with three clockwise stitches using Prolene 2-0. Hiatoplasty was also performed using Prolene 2-0 stitches facing the posterior pillars, and a final stitch with Prolene 2-0 was placed from the stomach to the right pillar. The patient went home the day after surgery without complications.

## Discussion

A true short esophagus remains an exclusive intraoperative diagnosis, even after a complete preoperative evaluation of the patient. Its relevance lies in the increase in recurrence risk in patients subjected to Nissen fundoplication (NF) [1]. In our institute, we routinely perform esophagogastroduodenoscopy, esophagrams, and CT scans in patients evaluated with indications for surgical correction of GERD/hiatal hernia. While we can attest to the complexity of the case with this image evaluation, the true diagnosis of short esophagus will only be obtained with intraoperative direct visualization. CG consists of the creation of a neoesophagus and the resection of a portion of the gastric fundus below the gastroesophageal junction [3]. The identification of the esophageal-gastric junction (E-G) is a key step in the surgical correction of hiatal hernia. During laparoscopy, it is possible to miss the exact position of the E-G junction because the proximal stomach attracted upward acquires a tunnel-like form after years of herniation, the serosa loses brightness, and the wall thickens. The tubularized proximal stomach is hardly distinguishable from the distal esophagus [4].

In patients with a short esophagus, the possibility of performing the Collis-Nissen procedure on a fundoplication should be considered to guarantee the sufficient length of the intra-abdominal esophagus for the adequate reconstruction of the reflux barrier and hiatoplasty, coupled with the high incidence of reconstruction failure and postoperative symptoms [2]. Among the most worrying complications is a possible leak from the staple line, as well as aperistaltic neosophagus and acid secretion above the gastroesophageal junction.

Although there is limited literature evaluating the long-term morbidity and mortality associated with CG, Lugaresi et al. compared patients undergoing CG with those undergoing NF. They assessed postoperative mortality during the first 30 days and morbidity over the first five years using clinical questioning, barium swallow, endoscopy, and manometry. They reported a mortality rate of 1.5% (one patient) due to CG fistula, with a major complication rate of 24% and a minor complication rate of 7%. Minor complications included pleural empyema without fistula, atrial fibrillation, acute pancreatitis, pneumonia, and severe dysphagia due to *Candida albicans*. The conversion rate was 6.15%. In the comparison of patients undergoing CG versus NF, no statistically significant differences were found in terms of morbidity, mortality, postoperative results, dysphagia, or dyspepsia between the two procedures [1].

Montcusi et al. evaluated patients with type III-IV hiatal hernia and a short esophagus undergoing CG for five years, including 80 patients. They reported an intraoperative complication rate of 5% and a postoperative complication rate of 21%, which included gastroparesis, seroma, acute urinary retention, lipothymia, postoperative ileus, empyema, and cellulitis with an abscess. The recurrence rate was 8.8%, with a notable improvement in preoperative dysphagia. They concluded that CG is a safe surgical technique with a low recurrence rate and significant improvement in preoperative dysphagia [5].

Recent literature suggests similar morbidity and quality of life outcomes between patients selected for the Collis procedure and those who required only the Nissen procedure. Additionally, there are significant, long-lasting reductions in hernia recurrence, heartburn, dysphagia, regurgitation, and antiacid medication intake [6-8].

## Conclusions

A short esophagus remains a controversial topic. Some authors argue that it is more frequent than suspected and responsible for high recurrences in specific patient groups. In this particular case, we consider the possibility of this scenario because of the evolution of the pathology and the presence of an enormous type IV hiatal hernia involving the transverse colon. Further analysis and studies are required to determine predictors of short esophagus in patients subjected to antireflux procedures, assisting surgeons who perform high-grade gastroesophageal surgical interventions.
